# Modified forced expiration technique using expiratory resistance in adults with cystic fibrosis

**DOI:** 10.1111/crj.13708

**Published:** 2023-10-04

**Authors:** Kathryn Watson, Emma Koenig, Ashleigh Bannister, Virginia Mayne, Angela Jacques, Abbey Sawyer, Jamie Wood

**Affiliations:** ^1^ Physiotherapy Department Sir Charles Gairdner Hospital Perth Australia; ^2^ Physiotherapy Department Fiona Stanley Hospital Perth Australia; ^3^ Department of Research Sir Charles Gairdner Hospital Perth Australia; ^4^ Department of Rehabilitation and Human Performance Icahn School of Medicine at Mount Sinai New York New York USA

**Keywords:** airway clearance, cystic fibrosis, positive expiratory pressure

## Abstract

**Background:**

Participation in airway clearance techniques (ACT) is important to minimise development of irreversible airway obstruction in patients with cystic fibrosis (CF). Positive expiratory pressure (PEP) and expiratory resistance devices (ERD) are often used as they can improve collateral ventilation and aid in the shearing of mucus from airways. This project looked to identify whether utilising an ERD during a forced expiration technique (FET) improves ease of expectoration, sputum amount and coughing frequency in patients with CF.

**Method:**

Patients with CF admitted for a respiratory exacerbation completed two sessions of 10 cycles of their usual ACT with half of the FET components performed with an ERD, half with FET alone.

**Results:**

EOE, sputum wet weight, cough frequency and patient preference were similar between groups. Improved EOE without the ERD was found in participants who usually use PEP for their ACT regime.

**Conclusion:**

Combining the FET with a PEP mask did not improve EOE and other outcomes in this small study. Investigating the efficacy of this technique within a larger population is warranted.

## Graphical abstract

This project looked at the impact of including an expiratory resistance device (ERD) during the forced expiratory component of an airway clearance regime in patients with CF. Ease of expectoration (EOE), sputum wet weight, cough frequency and patient preference were similar between groups. Improved EOE without the ERD was found in participants who usually use PEP for their ACT regime.

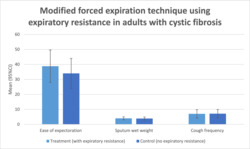

## 1 INTRODUCTION

Airway clearance techniques (ACT) are a cornerstone of treatment for people with CF, reducing airway obstruction and slowing disease progression.^1^ No one technique is more effective,^2^, ^3^, ^4^ with guidelines recommending that treatment should be individualised.^5^, ^6^ Given the high energy expenditure associated with completing ACT,^7^ and fatigue a reported barrier to adhering to ACT regimens,^8^ the goal should be to clear the most amount of sputum with the least effort.

Positive expiratory pressure (PEP) is a commonly used ACT, facilitating clearance by promoting collateral ventilation^9^ and splinting airways during expiration.^9^ While the latter is not the primary mechanism, it has been implemented in individuals with tracheomalacia.^10^

The forced expiration technique (FET) is commonly regarded as the most effective part of ACT,^11^ and combines forced expirations (huffing) with periods of breathing control. When performed correctly, expiratory flow is increased, mobilising secretions towards the central airways.^12^ When performed too forcefully, it can result in airway closure and excessive coughing. The addition of a PEP mask during the FET may improve effectiveness and ease of expectoration (EOE).

The objective of this trial was to evaluate whether performing the FET with a PEP mask improves EOE and other outcomes in adults with CF.

## 2 METHODS

This was a pilot randomised crossover trial (ACTRN12619000224123). Participants were recruited from the respiratory ward at Sir Charles Gairdner Hospital. Inclusion criteria were being admitted with a respiratory exacerbation of CF and aged at least 18 years. Individuals were excluded if they had current haemoptysis; were using non‐invasive ventilation for ACT; had a pneumothorax; or were unable to expectorate sputum.

Participants completed two treatment sessions on the same day consisting of 10 cycles of their usual ACT. They were randomly allocated to perform the FET with or without the PEP and mask at the end of the first cycle of ACT and then alternated the use of the PEP with the FET at the end of each subsequent cycle. This was repeated in the afternoon session; however, the first cycle started with the opposite FET method to the first cycle in the morning session.

The primary outcome of the study was EOE, measured using a 10‐cm Visual Analogue Scale (VAS) ranging from easiest possible (0 cm) to hardest possible (10 cm), recorded at the end of each ACT cycle by a blinded assessor.

Secondary outcomes were sputum wet weight, cough frequency and preference. Expectorated sputum was measured by a blinded assessor following each session. Cough frequency in each cycle was manually counted by the physiotherapist with coughs counted as single cough epochs to account for clusters of coughs.^13^

Power calculation was based on a study using the same outcome measure in adults with CF^14^ with an aim to show a between‐group difference of 1.0 cm on the VAS. Based on 10 repeated measurements, the required sample size was eight per group. Additional participants were recruited to account for any incomplete participant data with all data included in the final intention‐to‐treat analysis.

Descriptive statistics were reported using Stata Version 17. Categorical comparisons between preference groups were performed using chi squared tests. Generalised linear mixed models with random subject effects, and adjusting for lung function, preference and order, were used to examine outcomes between intervention groups.

## 3 RESULTS

Fifteen participants were entered into the study, with complete data available for 12. Baseline variables were taken from inpatient notes (Table 1). There were incomplete data for three participants: one excluded due to haemoptysis occurring during the morning session and two due to participants feeling unable to provide accurate VAS responses.

**TABLE 1:** Baseline characteristics.

Baseline characteristics	*n* (%)
Demographics	
Age^a^	24 (21, 32) [19–59]
Sex female	7 (47)
BMI < 18	2 (13)
Day of admission data collected^a^	5 (3.5, 7.5) [1–12]
Length of hospital stay (days)^a^	15 (10, 15) [3–19]
Genotype	
Homozygous F508del	7 (47)
Heterozygous F508del	6 (40)
Other	2 (13)
Modulators	
Ivacaftor	1 (7)
Symdeko	2 (13)
Nil	12 (13)
Usual ACT	
Aerobika + HTS 3%	1 (7)
Aerobika + HTS 6%	3 (20)
Aerobika + NS	1 (7)
Flutter	1 (7)
Flutter + HTS 6%	1 (7)
PariPEP + HTS 6%	6 (40)
PariPEP	1 (7)
PEP Mask + HTS 6%	1 (7)
Dornase‐alpha prescription	
Once daily (≥45 min prior to AM session)	4 (27)
Twice daily (≥45 min prior to AM session and post PM session)	10 (67)
Spirometry	
Current FEV1^a^	1.90 (0.98, 2.60) [0.67–4.25]
Current FEV1%^a^	52 (28, 70) [24–87]
Current FVC^a^	3.02 (1.83, 4.05) [1.24–6.04]
Current FVC %^a^	70 (42, 76) [1–96]
Pseudomonas colonisation	14 (88)
# exacerbations in 12 months^a^	2 (2, 3) [1–5]
Haemoptysis in 12 months	3 (20)

*Note*: Data summarised as *n* (%) unless specified otherwise.

^a^ Med (IQR) [min–max].

Results for all outcomes are included in Table 2. There was no difference in EOE between groups [mean difference (MD) 4.53, 95% CI −0.29–9.36, *p* = 0.06]. Subgroup analysis stratifying for usual ACT identified a difference in EOE between groups favouring the control (i.e. FET without PEP) in those who usually use PEP for their ACT regime (MD 10.69, 95% CI 2.24–19.140, *p* = 0.01).

**TABLE 2:** Results.

	Control (no mask) mean (95% CI)	Treatment (mask) mean (95% CI)	Est. mean difference (95% CI)	*p*
EOE	34.0 (23.9–44.0)	38.8 (28.0–49.5)	4.5 (−0.3–9.4)	0.06
AM session	34.6 (24.0–45.2)	40.2 (28.8–51.6)	5.6 (−1.5–12.7)	0.12
PM session	33.2 (22.8–43.6)	37.3 (26.2–48.3)	4.0 (−2.9–10.9)	0.25
EOE (usual ACT = aerobika)	43.4 (39.9–46.8)	42.0 (38.6–45.5)	−1.3 (−6.2–3.5)	0.59
AM session	43.1 (38.2–48.0)	42.0 (37.1–46.9)	−1.1 (−8.0–5.8)	0.75
PM session	43.7 (38.8–48.6)	42.1 (37.3–46.9)	−1.6 (−8.4–5.3)	0.65
EOE (usual ACT = PEP)	31.5 (21.6–41.3)	42.1 (30.7–53.6)	10.7 (2.2–19.1)	0.01
AM session	31.6 (20.1–43.0)	44.7 (31.2–58.2)	13.1 (1.2–25.1)	0.03
PM session	31.1 (19.8–42.5)	39.3 (26.4–52.2)	8.2 (−3.4–19.8)	0.16
Sputum wet weight	3.9 (2.9–4.8)	4.0 (3.0–5.0)	0.1 (−0.3–0.5)	0.60
AM session	3.7 (2.7–4.7)	4.1 (3.1–5.1)	0.4 (−0.2–0.9)	0.16
PM session	4.0 (3.0–5.1)	3.9 (2.9–4.9)	−0.2 (−0.7–0.4)	0.51
Cough frequency	7.1 (4.3–9.9)	7.0 (4.2–9.8)	−0.1 (−0.3–0.1)	0.88
AM session	7.4 (4.3–10.5)	7.9 (4.6–11.2)	0.5 (−1.2–2.2)	0.55
PM session	6.8 (4.0–9.7)	6.1 (3.6–8.7)	−0.7 (−2.1–0.8)	0.37

*Notes*: Outcome comparisons, adjusting for order, FEV1 and FVC and including random subject effects. FCEOE models adjusted for usual ACT *p* = 0.064, adjusted for preference *p* = 0.056.

Abbreviations: ACT, airway clearance technique; EOE, ease of expectoration; PEP, positive expiratory pressure.

There was no difference in sputum wet weight or cough frequency between groups overall or when stratified for usual ACT (MD 0.10, 95% CI −0.29–0.49, *p* = 0.60 and MD −0.08, 95% CI −0.31–0.14, *p* = 0.88, respectively).

Seven (58%) participants preferred FET without the mask. One participant changed preference from the AM to PM session. Of the participants who preferred no mask, 67% usually used PEP for their ACT. Of those who preferred using the mask, 46% used PEP as their usual ACT, and 46% used Aerobika. There was no difference for preference between treatment groups (*p* = 0.55).

## 4 DISCUSSION

This study investigated the impact of adding expiratory resistance to the FET in adults with CF experiencing an acute exacerbation and found no difference in EOE, sputum wet weight, cough frequency or preference.

Despite the known benefits of PEP splinting airways open during expiration, improving airflow and secretion removal,^9^ no benefit was observed when adding PEP to FET in this study. It is probable that the power of the study was inadequate to demonstrate a change, with complete data available for just 12 participants.

The trend towards improved EOE without the PEP mask may have been influenced by the use of PEP as usual ACT. Subgroup analysis identified better EOE within the control intervention in participants who usually used PEP for their ACT (*n* = 8), potentially due to the PEP already improving EOE when used for the ACT cycle.

The lack of differences in other secondary outcomes is likely due to the low power of the study and inability to blind participants to the intervention. It is not clear whether the EOE experienced led to a decision about preference or whether an individual's preconceived notion of using a mask for the FET component determined the EOE. Only a small number of participants (three) were using modulator therapy; therefore, this was unlikely to have influenced outcomes.

Another limitation was using wet sputum weight due to the diluting effect saliva can have on sputum, but sputum wet weight is an accepted simple and timely way to measure airway clearance.^15^

This is the first work looking at the impact of combining PEP mask with FET during ACT. Findings from the present study align with recommendations from previous systematic reviews that treatment choices should be based on individual preference and individualised outcomes.^5^, ^6^

## 5 CONCLUSION

Combining the FET with a PEP mask did not improve EOE and other outcomes in this small study. Investigating the efficacy of this technique within a larger population is warranted.

This work received ethics approval and consent to publish through the Sir Charles Gairdner and Osborne Park Health Care Group Human Research Ethics Committee, PRN RGS0000000062.

The data that support the findings of this study are available from the corresponding author upon reasonable request.

## AUTHOR CONTRIBUTIONS

All authors contributed to the research design and wrote the paper; Kathryn Watson, Emma Koenig, Ashleigh Bannister and Virginia Mayne performed the research/collected data; Angela Jacques and Jamie Wood analysed data.

## CONFLICT OF INTEREST STATEMENT

All authors declare no conflicts of interest.

## ETHICS STATEMENT

This work received ethics approval and consent to publish through the Sir Charles Gairdner and Osborne Park Health Care Group Human Research Ethics Committee, PRN RGS0000000062.

## Data Availability

The data that support the findings of this study are available from the corresponding author upon reasonable request.
